# Application of Artificial Teeth, by the Auroplastic Principle

**Published:** 1853-10

**Authors:** Edwin Truman

**Affiliations:** Dentist, London.


### SELECTED ARTICLES.

### ARTICLE XIV.

### *Application of Artificial Teeth, by the Auroplastic Principle.* By Edwin Truman, Dentist, London.

It is necessary to preface the subject of the present paper with a brief description of the means now in use for supplying the deficiencies of teeth; the ill effects of which must be apparent to all—more especially to the medical man, who knows the great part they play in the functions of mastication and enunciation. In the former, their loss subjects the stomach to double labor; not only from the food not being sufficiently comminuted, but being imperfectly insalivated thereby. In the latter function, the tongue, deprived of the wall against which it presses in the different modifications of the external opening for sound, is not able sufficiently to compress itself or lessen the cavity of the mouth whereby these modifications are produced, and so causes the pronunciation to be thick and obscure; especially in the quick changes that are necessary in singing or public speaking.

Until within the last fifty years, we may fairly say, that the construction of artificial teeth was not at all understood. I shall not, however, give any history of the past in this matter; but merely describe the present methods, in order that I may be more clearly understood when treating of my improvements.

They were originally made in a most rude and uncouth fashion; there was scarcely any attempt, in many instances, to imitate nature even in shape. Forgetful entirely of utility—the truly essential point in their construction, the desideratum of all good dentists—they seemed rather intended to fill the mouth than to aid in mastication. Previously to that period, the frames and sockets were constructed of the same material as at present, with natural or bone teeth fitted to them. These plates were either tied into the mouth with ligatures of silk or silver wire, or clasps were placed in such a manner as to clip the other teeth. I need not say how detrimental such a mode of proceeding must have been, if not performed with the greatest precision and delicacy. I have even in my time seen teeth cut through by the pressure of such bands of support. Thanks to many of the enlightened practitioners of the present day, these methods are either exploded or performed much more accurately.

There is no one method that can, as a general rule, be laid down for the construction of artificial teeth. The plan adopted must depend on the peculiarities of the case; but, with all improvements, there are defects of great magnitude still existing, both in the method of construction and in the materials employed. I shall, therefore, enumerate the different modes now in use, and show the weak points in each. The number of processes necessary to go through in constructing a set of teeth renders it a matter of the greatest uncertainty as to its ultimate fit; besides which, the unyielding materials of which it is made are not at all calculated to produce that elastic solidity, if I may be allowed the expression, that is so essential to their comfort and utility. I do not in this include the teeth themselves, as the harder and more durable they are the better. In fact, I may say that in my following remarks I allude exclusively to the bed or sockets that come in contact with the gum. As for the teeth themselves, I prefer the mineral ones; better substitutes than which it is, I believe, impossible to obtain. Thanks to Mr. Ash and others, we have in that material a most beautiful and delicate imitation of nature, with all that transparency so characteristic of the finest productions of the human gums, without their defect of being subject to decay.

The present method of manufacturing artificial teeth is first to take a model of the mouth in wax. This is done by pressing wax of a right consistence upon the gums to be modeled; and it may, with care, be accomplished with the greatest nicety in all those cases where the outlet for the wax is larger than, or as large as, the surface to be modeled. But if the teeth overhang the gum in such a manner as to lessen the space originally existing between the crowns, or, in other words, the tooth behind the deficiency and that in front slope towards each other, as very often happens in the under jaw, then the wax cannot be withdrawn perfect. There are many other circumstances under which a good model cannot be obtained—smallness in the external opening—extreme tendency to nausea—malformation—and many other causes. In these cases the best model is obtained that it is possible to procure, and any imperfection that may exist in it allowed for to the best of the skill of the operator in the next process, which is that of producing a cast of the mouth from the wax model.

This is done by throwing plaster of paris into it, and, on removing the wax, there remains its exact counterpart, which from a good impression, is a fac-simile of the surface to be fitted. But, if circumstances have prevented the operator from procuring a perfect model in wax, then he must alter the plaster until it is like the mouth. This is a most difficult operation, and one that can seldom be done well; it is the hindrance to a good fit in hundreds of cases; but it is the only alternative that is left to the dentist, and in the method or dexterity with which he performs it lies his skill.

It will be instantly seen by every body that, in this stage, certainty is lost in some degree; but we have another stage yet to go through. If the gold frame is to be used—and, on account of its durability and cleanliness, it is the material most generally adopted—the dentist is necessitated to recast it in metal, the plaster not being hard enough to work the metal plate on. Here all our difficulties occur over again, with the addition of one or two minor ones, which I do not particularly mention, as I do not in the least wish to lay claim to rectifying any defect that does not really interfere with the possibility of constructing artificial teeth comfortably and durably with our present means, and great skill in the operator. There are many practitioners of the present time quite competent to deal with any obstacles that are not insurmountable—such as are those I enumerate—by the present system.

The gold plate is afterwards struck between this metal model and a soft metal die to fit to the indentations and peculiarities of the mouth; and this can be done with great nicety by a good workman. But it is a difficult stage: first, from the contraction of the metal more or less in cooling, if the model be not cast in an alloy that is little affected by temperature, an exact fit can scarcely be hoped for. To obtain this, we are obliged to use the softer metals, as bismuth and tin: and, should these not be in the right proportions, and used for some time, the model is very apt to be injured in the process of striking, as considerable force is necessary to bring up the plate to a sharp and close fit. The model is, also, very apt to spread under the blow; and should this happen, ever so slightly, it prevents a firm fit, causing the plate to press unequally, and to rock or tilt in the mouth, thereby producing considerable pain and irritation, as it brings pressure laterally against the gum, instead of perpendicularly where the breadth of surface allows it to be borne.

The plate being thus far produced, the next process is to adapt it so in shape around the remaining teeth, that it shall hold its position during the action of the mouth. This is an operation that the dentist would willingly be able to dispense with for many reasons. First, the danger of injuring the remaining teeth; then the difficulty of so placing them that the gold shall not be visible—and often the solidity is sacrificed to effect this.

Several very ingenious methods are in use to do this without the assistance of the remaining teeth. One of these is to sink hollows in the under surface of the plate to assist adhesion by producing a vacuum; engraving, also, has been recommended: but there are few cases that succeed by either plan, especially the latter. I never found the former of any use, but Mr. Lemail tells me he has used it with great success. There have been other expedients made use of, but my space will not allow me to mention any but those most generally practiced and recommended.

The artificial teeth are then fastened to this gold plate by gold pins, attached in such a position as to allow the new teeth to occupy the position of those that are lost. This renders the case complete. If much gum have been lost, or in other words, if the subjacent structures are much absorbed, then we have, until very lately, been unable to produce a substitute for them in any material that is not very soon rendered defective by its decomposition. Ivory stained was the only thing in use. Mineral paste has been tried, but never has been brought to any degree of perfection till very lately. Mr. Ash, to whom we must at all times pay the tribute due to his improvements in almost all things used by the dentist, has produced some of his beautiful mineral teeth with a mineral gum attached, which will, in many instances, remedy the above defect to a great extent.

Before leaving this part of my subject, I would observe that several attempts have been made to line the gold plates with soft material, to relieve the gums from hard pressure. The last that has come under my notice, is Mr. Saunder's plan of using india-rubber. After moulding this substance to the inner surface of the plate, which he had previously coated with silver, he caused it to be vulcanized: the sulphur used in this process entered into combination with the silver and formed sulphate of silver, which fixed the india-rubber to the plate. Mr. Saunders does not, however, use this plan now, as it was found that the saliva dissolved the sulphate of silver, and the caoutchouc became detached. This, however, was a step in the right path; and, by showing us what may be done, furnishes us with a good guide in our future experiments.

The next method of supplying artificial teeth is by ivory, or the tooth of the hippopotamus, &c., so carved as to fit the gum. This may be done, with care, on the plaster model, and the necessity for metal casting obviated. But its great defect is its want of durability, which causes it to be dispensed with, if possible. It is a process attended with more certainty, perhaps, than the other: but the defects of wax models are still existing. The plaster is rendered hard by resin, &c., and the ivory is carved to fit it. The teeth are then let into their proper places, and the gum is formed out of the ivory in the usual way.

The plate or bone is kept in its place by little clasps of gold wire round the remaining teeth. These cannot often be dispensed with—at any rate, at first; but, after wearing them for some time, the ivory becomes softened by the action of the saliva, and from this time, until the decay positively becomes apparent, they are the most comfortable artificial teeth now in use; but unfortunately, this is a short time, in most cases not more than twelve months, and then all our work has to be done again.

All the other plans of fitting artificial teeth now in use, are modifications of these two. The usual and best mode, in the general run of cases, is to use the gold plate for the upper jaw, and the bone for the under.

I have now to speak of the greatest defect in artificial teeth. I allude to the spiral spring used to retain the upper plate in its position, in cases where there are no teeth remaining, and the gum is so flat as to prevent their holding by suction: which is nearly always the case; at any rate, at the commencement of their use. The two sets are connected by a spiral spring on each side the jaw, attached to the under and upper set by each end, and, by its action and tendency to preserve a straight line, keeping the sets pressed upwards and downwards. These springs have been and still are a source of very great annoyance to the wearer; for, fit them ever so nicely, they rub against the cheek—they prevent the free use of the tongue—they collect the food around them; but their greatest defect of all is the increased pressure on the under jaw, a part that is of all others the least able to bear it, especially, if the artificial work touching the gum is of the hard, unyielding material now in use. This is the reason for always using ivory in the under cases; but its non-durability and the offensive odor it is apt to give the breath when in a state of decay often tempt dentists to construct these under plates of gold, and then it is that the annoyance of the springs is felt the most, especially, if they be not fitted with the utmost nicety and success.

To show the reason why hard substances produce the ill effects they do, and to show what really is necessary in the construction of artificial teeth, I must be permitted to say a few words on the construction of the parts of the mouth that come under pressure of, or in contact with artificial teeth.

The teeth are set by nature in bony sockets fitted for their reception, formed by the alveolar processes of each jaw. These are covered by many structures of great susceptibility: first, the periosteum, with its vessels and nerves; then the gum—this is not a very sensitive structure, fortunately; but it is covered by the mucous membrane of the mouth, and its accompanying delicate structures, the vessels and nerves of touch, which, by pressure, become inflamed. On the loss of a tooth, the alveolar processes become absorbed, the socket filled up, and the soft structures adapted to cover the now unyielding and solid jaw: this process does not always proceed at the same rate or in the same equal manner. Sometimes the surface left under the gum is equal and smooth—at other times, unequal and in deep hollows; the gum and parts above are not of uniform thickness, so that uniform pressure is very difficult to obtain—especially, as in modeling, these soft parts are only pressed between the hard bone and soft wax, whereas, afterwards, they are pressed between two hard substances necessarily unequal from the unequal thickness of the soft parts under the different circumstances. If the surface be large, then the inconvenience is not so much felt, as the pressure is distributed, and thereby lessened on each individual part.

But in the under jaw, from its form, this extended surface cannot be obtained; and here, to add to our difficulties, the absorption always leaves the upper surface of the bone more or less angular, sometimes forming quite a sharp bony ridge, with a very thin coating of gum between it and the mucous membrane, whilst, close to it, on the inner side, are the soft structures under the tongue and folds of mucous membrane, the slightest pressure on them being almost insupportable. On the outer side, we have only the mucous membrane and gum, and here, therefore, we are obliged to rear our structure; but as from the form, the plate laterally will be nearly perpendicular, and as the motion of the cheeks must be interferred with as little as possible, we have little space left. The lower surface is, therefore, at its widest part very narrow; and any extra pressure, from springs or other causes, especially mastication, presses the perpendicular edge of the plate against the soft parts, and sets up inflammation and its attendant consequences.

It appears, from the foregoing remarks, that an entirely new material should, if possible, be found for the construction of the plate, either alone, or in conjunction with those already in use, to relieve the pressure on the soft structures of the mouth; and, at the same time some method should, if possible, be adopted to procure *certainty* in the fit, combined with solidity and durability in the material. The substance used must be pure, and free from any evil or injurious effect on the structures of the mouth—it should not be hard, or rather, not much harder than the gums themselves—it should be capable of resisting the solvent properties of the saliva and acid, and should be easy of manipulation. Such a substance we possess in gutta percha, and I purpose to show the method by which this substance may be used in the construction of artificial dentures, and be made the means of remedying the greatest defects now existing in them, from its possessing many qualities combined that are not to be so found in any other substance. I shall first point out its peculiarities; not that it is a substance now unknown or unappreciated, but because I think I shall show it to possess qualities that eminently fit it for the purpose of the dentist. It has been long in use for filling teeth, and has been used in the construction of false palates, therefore its qualities in the mouth have been well tested, even in its impure state, as until very lately the pure white gutta percha could not be obtained. This I presume to have been the reason why none of my professional brethren have yet introduced it into the manufacture of artificial teeth. I have, since its first appearance in this country, been deeply interested in it, and have tried innumerable experiments with it. It is, at present, little appreciated, compared to what it will be—and I have no hesitation in saying that a tithe of its capabilities are not known, and that it will be turned to uses, compared with which, in importance, its applications hitherto are as nothing. I will now enumerate some of its most important qualities and peculiarities, and show in what way they serve the dentist; and, in conclusion, I shall briefly detail the mode by which I intend to apply its usefulness to my art.

First, as to its purity. This must of course be our first consideration, and in considering this part of our subject, there are many points requiring attention. Is it capable of decomposition, producing an unhealthy influence? Does it become softened and offensive? These questions must be answered before we can apply it to our use. Is it a substance that can be retained in the mouth for a long time without detriment to the health of the patient, or injury to the surrounding tissues?

It is in its pure state one of the most indestructible substances known, and in cases under my own observation, has been worn for years in the mouth without in the slightest injuring either the general health of the patient, or the tissues that come in contact with it. I have used it now many years; and although it does not wear the same in all mouths, yet in none have I found the slightest ill effects. On the contrary, the mouths of patients having gutta percha palates are so little inconvenienced by it, that after it is removed, it is impossible, upon examination, to tell that any artificial work had been there. This is not the case even with bone; and with gold worn for any length of time, a distinct mark is left upon the gum, the whole of the surface covered by the plate being a darker red than the rest. This is, perhaps, no very great defect; but I mention it as a proof of the inoffensive nature of gutta percha, in the use of which substance, it never happens. Bone worn in the mouth sooner or later decays, and in so doing, becomes black, soft and offensive, and must be replaced, the whole structure being useless. When durability therefore is a matter of consequence, gold must be employed instead; as it is in all cases more or less. No two cases fit the mouth equally comfortably and well; and nothing is more annoying, after a set of teeth have become comfortable and the mouth accustomed to them, than to find from decay that they are worn out and a new set requisite, which, like new shoes on tender feet, must be worn some time before they are easy. These defects do not exist with gutta percha; it does not decay in the mouth, and, if it did, it would not be accompanied by the same disagreeable consequences as ivory.

I have had some cases worn nearly three years, that remained the same as in the first hour they were made. Others, by the friction of the mouth and food, become slightly abraded at the edges; but never softened, never inherently offensive. When any offensive odor does exist, it is from foul breath, want of cleanliness, or from diseased stumps, which the patient has refused to have extracted, (an operation, it is not in the least necessary to undergo, when gutta percha is used.) Experience, therefore, has fully answered the question of its purity. There is, however, one more advantage: even should it wear away, it does not spoil the case, since it can be replaced at one sitting, at any time, and that without in any way altering the old comfortable artificial teeth to which the mouth has become accustomed, and this can be repeated to any extent.

Next, its *specific gravity* is very low, so that any structure composed of it, however large, is light, in comparison to what it would be, constructed either of ivory or metal. This is of great consequence to us, as the lighter our artificial teeth are, the better, providing we have sufficient strength.

This brings us to its *solidity;* and here would appear, at first sight, to lie its weak point, instead of which we have its greatest excellence. It will not alter its shape at any temperature under 100, whilst at 20 degrees higher temperature, it may be moulded to the most intricate shape, with the greatest facility. At a low temperature it is hard and unyielding, and at the usual temperature of the mouth, it is sufficiently solid to retain its shape under any extent of pressure, and yet too soft to injure the structures on which it presses. Its great elasticity, too, allows it to yield to resistance, only to return to its exact form again when the resistance is removed. It will sustain great friction with but slight effect, we may almost say, with impunity, although, at the temperature of the mouth, the fine filaments on the surface become slightly raised by friction. I shall presently show my plan for obviating this, which, in some cases, would be a defect. I think it right to mention all its defects as well as good properties, that the knowledge of them may assist my fellow-laborers in their future experiments on this substance when used in the mouth.

Its *durability*, under the circumstances in which we place it, is very great. It is soluble in scarcely any thing but strong spirits, as ether, naptha, or chloroform. It is quite insensible to the effect of acids, and the strongest alkalies have no more effect. It is quite impervious to that great solvent, water; and, although I have had it in the mouth, where heat and moisture combine under the most favorable circumstances for decomposition, for years, I have not found the least sign of decay.

*Color.*—If the white gutta percha be taken for the base, it can be colored to the greatest nicety of tint. But here is another defect, and, I think, its greatest; light affects it, and I do not think any color, unless brown, perhaps, will remain long uninjured, if subjected to light, as, like caoutchouc, light has power of turning it brown, the color that is seen in general use. In the mouth, this does not happen to any extent, as mostly the mouth is dark, and those parts forming the gums, I shall show, should be covered with certain substances to protect them.

*Fine Texture.*—This is a quality pre-eminently possessed by this substance, and one that is of the greatest consequence to us. I am told, that since its introduction, it has been used by seal engravers, to try their impressions on, in preference to sealing-wax, as it gives a finer impression. I have a piece that I moulded to the side of a shell, that has the prismatic colors transferred to it, which, I think, as great a proof as can be given of its capabilities in this respect. In use in the mouth, the impression is far more beautiful than in wax, the slightest peculiarity of texture in the mucous membrane being represented.

I shall now proceed to give a detailed account of my plan for availing ourselves of the advantages of this material. I shall follow the same plan here that I pursued in my discussion of the artificial teeth now in use; give the process of manufacture, stage by stage, until we have the complete artificial teeth. I shall then show the method of adapting them to the mouth, *after they are finished*, and how we can regain any certainty that we have lost in manufacture, and, in conclusion, say a few words in comparison of the different methods.

First, I take the best model that I can obtain in wax, in the usual way, and prepare a plaster cast from it, in which I do not attempt to rectify any defects, as that can be better done in a future process; this plaster cast I render as hard as possible, with resin and wax in equal proportions, which renders the plaster impervious to water, and enables me to immerse it in cold water with impunity.

This being completed, I construct a gold wire frame in such a manner as to support the different artificial teeth firmly in their places, and, likewise, give strength to those parts where the gutta percha is necessarily thin, as behind the front teeth in the lower jaw; this frame I firmly solder together, rendering it alone as strong as the complete structures on the old plan; but I construct it in such a manner, that in the finished teeth it is embedded in the centre of the gutta percha, and no part of it allowed to touch either the gums or remaining teeth, its only object being to give firmness and strength, and to attach the teeth.

I then proceed to mould gutta percha around this frame, on the plaster model, in such a manner as to produce the shape required for replacing all the deficient structures of the mouth, placing the mineral teeth already prepared on the pins fixed for their reception, and moulding the gutta percha around them, so as to replace the sockets and gum originally existing around the natural teeth; this structure may then be finished to such a nicety as to represent accurately every peculiarity of surface.

Then I place the work in the mouth at a temperature just sufficiently high to enable the gutta percha to be moulded to the mouth itself, and thus it receives the shape it is to retain until quite finished.

I then remove the mineral teeth, leaving the sockets that receive them empty, and the gutta percha on all sides apparent, preparatory to my last process; this being to cover all the surfaces that come in contact with friction, either from the tongue or cheeks, with a strong plate of gold, which may be obtained by the electrotype process; being particular not to have any gold in such a position that it can possibly touch the soft parts under, in pressure, during the action of the mouth, but allowing it to dip into every socket for the mineral teeth, thereby giving them great strength. The teeth are again replaced, and fastened on the gold pins in their sockets, and all that portion of the gutta percha that represents the gum, I either color by means of pigments, or enamel the surface of the gold which has been allowed to pass over it. This completes my artifical teeth, without one destructible or defective material being used, and yet without subjecting the gums to any hard, unyielding substance.

I then immerse them in water, at about 120 or 130 degrees, which accomplishes two objects; first, removes any unpleasant sensation from the cold metal; and secondly, enables the gutta percha to intimately adapt itself to its resting place, whilst the mouth is in action for the first few minutes, and so to receive from the mouth itself the form that it is to retain, and thus rectify any imperfection in the plaster cast or wax impression.

In conclusion, I have to compare the old system with the one I advocate. First, in the latter we rectify all uncertainty that existed in the former as to fit; next, we remove the pressure of hard substances, and equalize the pressure on the parts beneath; then we are able, from the exact fit obtained, to dispense with any fastening whatever around the remaining teeth—even in whole sets the spiral springs are not required. I have many whole sets on this principle, now in use, that are more firm without them than they have ever been with them on the old principle. The irregularities of the remaining teeth, that stood so much in our way, are of great use to us in the new system, as they tend to support and steady the artificial structure that we have been enabled to mould directly to them, without having recourse to those duplicate impressions so incompatible with certainty. In fact, we take nature's own plan in rectifying defects: the surrounding parts mould the new structures to suit themselves; whilst, in the old system, any alteration or allowance that took place after the artificial teeth were in the mouth, was affected by the new teeth on the mouth, instead of the mouth on the teeth; and I need not say which is the better.

For durability, I am sure it can compete with the old plan: and all those perplexing little accidents, so common in the best work now, will be prevented by the extra strength obtained, and the support given to the teeth by the new sockets. What I have shown will, I think, be apparent to all who wear artificial teeth, and even to those who are unaccustomed to their use. Of one thing I am confident, that none of my professional brethren will gainsay one word that I have uttered here, and that this method will be adopted in all those cases adapted to its use, which are innumerable.

I find, since writing the above, that some dentists have given an unfair report to their patients of my plan. Such a proceeding scarcely deserves comment; of all new plans, jealousies will occur: the majority, however, have given me a favorable decision; to some I have granted licenses, and done cases for others that they might test it more fully. I shall still be happy, in any way, to further their views, if practicable. Should the public ear be abused by unfavorable reports of my plan, I can only say, "See it and judge," which can at any time be done, without fee, by granting me the favor of a visit.

